# Evaluation of ^99m^ Tc-MccJ25 peptide analog in mice bearing B16F10 melanoma tumor as a diagnostic radiotracer

**DOI:** 10.22038/AOJNMB.2019.37712.1251

**Published:** 2019

**Authors:** Maryam Mazaheri Tehrani, Mostafa Erfani, Nour Amirmozafari, Taher Nejadsattari

**Affiliations:** 1 Department of Microbiology, Science and Research Branch, Islamic Azad University, Tehran, Iran; 2 Radiation Application Research School, Nuclear Science and Technology Research Institute (NSTRI), Tehran, Iran; 3 Department of Microbiology, School of Medicine, Iran University of Medical Sciences, Tehran, Iran; 4 Department of Biology, Science and Research Branch, Islamic Azad University, Tehran, Iran

**Keywords:** B16F10, Radiotracer, Tumor, ^ 99m^Tc-MccJ25

## Abstract

**Objective(s):**

Despite recent advances in treatment modalities, cancer remains a major source of morbidity and mortality throughout the world. Currently, the development of sensitive and specific molecular imaging probes for early diagnosis of cancer is still a problematic challenge. Previous studies have been shown that some of the antimicrobial peptides (AMPs) exhibit a broad spectrum of cytotoxic activity against cancerous cells in addition to their antimicrobial activities. MicrocinJ25 (MccJ25) is an antimicrobial peptide that is produced by *Escherichia coli* (*E. coli*) strain. The aim of this study was to investigate the potential of a new peptide radiopharmaceutical derived from MccJ25 for diagnosis of melanoma tumor bearing C57BL/6 mice.

**Methods:**

A 14 amino acid analog of MccJ25 was labeled with technetium-99m (^99m^Tc) through hydrazinonicotinamide (HYNIC) chelator and tricine as coligand. In vivo tumor uptake and tissue distribution were evaluated. The in vivo biodistribution studies were determined in C57BL/6 mice bearing B16F10 tumor.

**Results:**

The amount of non-peptide related ^99m^Tc-impurities that measured by thin layer chromatography (TLC) did not exceed 5% of the total radioactivity. The in vitro binding to B16F10 cells was 30.73 ± 0.9% after 1 h incubation at 37°C, and saturation binding experiments showed good affinity for radio-complex (K_d_=47.98±6.25 nM). The melanoma tumor was clearly visible up 1 h post-injection by gamma camera imaging.

**Conclusion:**

The results showed that ^99m^Tc-labeld peptide could be a promising candidate as a targeting radiopharmaceutical for melanoma tumor imaging in mice.

## Introduction

Nowadays, cancer is the second most common cause of death worldwide (1), caused by an abnormal cellular growth, in an uncontrolled manner, with the ability to invade other tissues, leading to the formation of tumor masses, neovascularization (angiogenesis), and metastasis (2).

Up to now, various peptide radiopharmaceuticals contain specific and non-specific radiotracers have been employed for tumor targeting and many types of cancerous cells based on positron emission tomography (PET) and single photon emission computed tomography (SPECT) imaging (3). Currently, ^18^F-2-fluoro-2-deoxy-glucose ([^18^F] FDG) as a glucose analog can be used for early detection of tumors in PET-imaging (4). However, [^18^F] FDG is not a tumor-specific imaging probe since it acts non-specific on the basis of increased glucose metabolism and unable to recognize tumors from non-malignant metabolically active processes in imaging (5). So, there is a critical need to develop novel effective imaging probes enabling early detection of tumors while at a curable stage.

Antimicrobial peptides (AMPs) have been implicated in tumor pathogenesis and have shown potential as novel anti-tumor agents (6). AMPs are biologically potent molecules of innate immune system with several roles that are found in all forms of life (7, 8). Some AMPs also show cytotoxic effects against diverse cancer cell lines, such as mouse myeloma, melanoma, lymphoma, leukemia, breast cancer, and lung cancer (9). In general, AMPs target the cell membrane of sensitive microorganisms and cancerous cells. They bind to negatively charged membrane surface by electrostatic interactions and disrupt the cellular membranes by various mechanisms and leading to cell lysis and death of cells (8- 10). Furthermore, some AMPs have anti- tumor properties via a direct cytolytic effect or the induction of tumor cell death, as well as chemotaxis and activation of immune cells, which mount tumor-related inflammatory responses (6).

MicrocinJ25 (MccJ25) is an antimicrobial peptide containing 21 amino acids and extensive post-translational modifications with a unusual lasso distinctive structure that is produced by a number of *Eeterobacteriacea* family especially *E. coli *(11, 12). MccJ25 is also a membrane-active peptide that induces the opening of mitochondrial permeability transition pores and the subsequent loss of cytochrome c and the death of eukaryotic cells (13, 14). MccJ25 also, due to its unique structure and high stability at extreme condition could be a valuable target for designing of different synthetic peptide analogs with high potential for use in nuclear medicine (15).

Recently our group chemically synthesized a 14 amino acid residue MccJ25 analog [cyclo (Cys^1^-Gly^2^-Ala^3^-Gly^4^-His^5^-Val^6^-Pro^7^-Cys^8^)-Tyr^9^-D-Tyr^10^-GABA^11^-D-Phe^12^-Tyr^13^-Gly^14^] using a standard Fmoc strategy. The evaluation of antibacterial and biological activities has been successfully performed and in vitro studies demonstrated the favorable biological characteristics for this peptide (16). To the best of our knowledge, there is no report in the literature on tumor imaging by a radiolabeled antimicrobial peptide derived from bacterial peptides. So, in this study, our efforts were continued for biological evaluation and localization of labeled peptide in mice bearing B16F10 melanoma tumor which is one of dangerous and aggressive forms of known skin cancers.

## Methods

2-chlorotrityl chloride [(2-Cl) Trt] resin and all of the Fmoc [N-alpha-(9-Fluorenylmethyloxycarbonyl)] protected amino acids were purchased from NovaBiochem (Laufelfinegen, Switzerland). Tricine (N-[tris-(hydroxymethyl) methyl] glycine) was obtained from Sigma (St. Louis, MO, USA). Other chemicals and solvents were purchased from major companies (Merck and Sigma-Aldrich) and used without further purification. ^99m^Tc-pertechnetate was obtained from a commercial ^99m^Mo/^99m^Tc generator (Pars Isotope Co). The cell culture medium RPMI 1640 supplemented with 10% fetal bovine serum (FBS), amino acids, vitamins and penicillin/streptomycin (Gibco, Eggenstein, Germany) was used for culturing of cell line B16F10. Analytical reverse phase high performance liquid chromatography (RP-HPLC) was performed on a JASCO 880-PU intelligent pump HPLC system (Tokyo, Japan) using a CC Nucleosil C_18 _column (250×4.6 mm Reprosil-pur ODS-3.5 μM) with the following gradient system of 0.1% trifluroacetic acid (TFA)/H_2_O as solvent A and acetonitrile as solvent B with a flow rate 1 ml/min and UV detection at 280 nm with a flow through gamma-detector containing a NaI crystal (Raytest-Gabi, Straubenhardt, Germany) and liner gradient of (0-100%) 0 min 95% A (5% B), 5 min 95% A (5% B), 25 min 0% A (100% B), 30 min 95% A (5% B) for 35 min. Mass spectrum was recorded on an Agilent 1100/Bruker Daltonic (Ion trap) VL instrument (LC-MS) using electro-spray ionization (ESI) mode. Quantitative gamma counting was performed on a well-type NaI γ counter EG&G/ORTEC Model 4001M Bin & Power Supply counters. Radioactive thin layer chromatography (RTLC) scanner (Raytest-GITA, Germany) was applied for radioactive-TLC experiments.

***Synthesis of HYNIC-peptide conjugate***

The protected peptide was synthesized by standard Fmoc strategy according to the reported procedure by our group (16), starting from 2-chlorotrityl [(2-Cl) Trt] resin with substitution 1.4 mmol/g. Briefly, coupling of each amino acid was performed in the presence of 3 mole excess of Fmoc-amino acid, N-hydroxybenzotriazole (HOBt), diisopropylcarbodiimide (DIC) and 9 mole excess of diisopropylethylamine (DIPEA) in dimethylformamide (DMF). Fmoc groups were removed by adding 20% (v/v) piperidine in DMF (20 mL). Protected peptide was cleaved from the resin by the cleavage solution containing 20% trifluoroethanol (TFE), 0.5% trifluoroacetic acid (TFA) and 0.5% water in dichloromethane (DCM) (20 mL) and incubated for 30 min at room temperature. The cyclization reaction was achieved by dissolving peptide in 10% aqueous methanol followed by addition of the iodine solution (380 mg I_2_ in 2 mL methanol). To indirectly radiolabeling of peptide with technesium-99m, Boc-HYNIC was coupled to the N-terminal of the peptide using 2.5 mol of 2-(7-aza-1H-benzotriazole-1-yl)-1,1,3,3-tetramethyluronium hexafluorophosphate (HATU) and 5 mol of DIPEA. De-protection of remained protecting groups was done by incubating for 1 h in a mixture of TFA, triisopropylsilane, thioanisole, and water (92.5:2.5:2.5:2.5). The crude product was precipitated into diethyl ether. Finally, the identity of the HYNIC-peptide conjugate was checked by electrospray ionization mass spectroscopy (ESI-MS) and the purity was assessed by analytical RP-HPLC.

**Table 1:** Analytical data of purified HYNIC-peptide conjugate

**Compound**	**Mass Spectrum**		**RP-HPLC**	
HYNIC-peptide	Calculated mass (g mol^-1^)	Observed mass (g mol^-1^)	Retention time (min)	Purity (%)
	1653.65	1654.5 [M+H]^+^ 1676.2 [M+ Na]+ 1696.4 [M+CH_3_CN+H]+	15.07	98.8%

**Table 2:** Biodistribution of labeled peptide in B16F10 tumor-bearing mice (% injected dose/g organ±SD, n=5)

**Organ**	**Time after injection**					
	**30 min**	**1 h**	**Block-1 h**	**2 h**	**4 h**	**24 h**
Blood	1.64 ± 0.03	1.05 ± 0.01	1.00 ± 0.02	0.76 ± 0.15	0.48 ± 0.09	0.05 ± 0.01
Heart	1.13 ± 0.22	1.01 ± 0.09	0.98 ± 0.05	0.84 ± 0.07	0.41 ± 0.05	0.07 ± 0.03
Lung	1.59 ± 0.20	1.34 ± 0.37	1.28 ± 0.30	1.24 ± 0.50	1.18 ± 0.40	0.12 ± 0.07
Bone	0.06 ± 0.05	0.67 ± 0.09	0.56 ± 0.07	0.64 ± 0.02	0.75 ± 0.07	0.11 ± 0.04
Stomach	0.83 ± 0.10	0.62 ± 0.15	0.60 ± 0.02	0.77 ± 0.09	0.81 ± 0.18	0.18 ± 0.06
Intestine	0.78 ± 0.06	0.84 ± 0.12	0.89 ± 0.10	0.84 ± 0.20	0.71 ± 0.06	0.50 ± 0.01
Thyroid	0.90 ± 0.12	0.10 ± 0.07	0.12 ± 0.04	0.25 ± 0.17	0.90 ± 0.04	0.07 ± 0.02
Liver	1.67 ± 0.05	1.55 ± 0.60	1.40 ± 0.80	2.02 ± 0.30	1.49 ± 0.20	0.35 ± 0.05
Spleen	1.76 ± 0.45	1.42 ± 0.30	1.03 ± 0.20	1.38 ± 0.04	0.77 ± 0.01	0.20 ± 0.10
Kidneys	1.91± 0.65	2.06 ± 0.96	1.98 ± 0.03	2.03 ± 0.08	1.55 ± 0.20	0.98 ± 0.30
Muscle	0.55 ± 0.08	0.49 ± 0.09	0.42 ± 0.05	0.35 ± 0.08	0.58 ± 0.09	0.02 ± 0.01
Tumor	0.97 ± 0.07	1.30 ± 0.12	0.78 ± 0.01	1.25 ± 0.24	0.80 ± 0.30	0.01 ± 0.02
Tumor/muscle	1.76 ±0.09	2.65 ± 0.03	1.85 ± 0.19	3.57 ± 0.01	1.37 ± 0.20	0.5 ± 0.01

**Figure 1:**
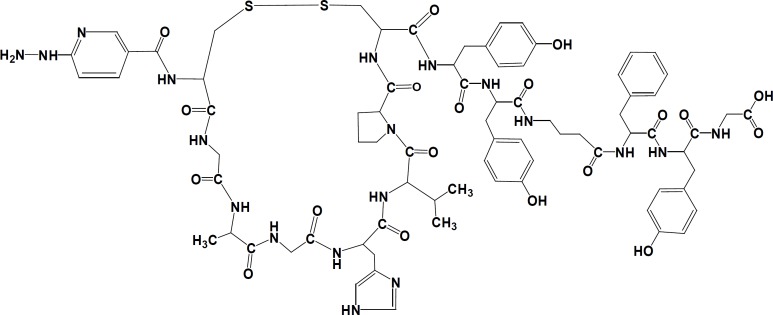
Chemical structure of synthetic HYNIC-peptide conjugate

**Figure 2:**
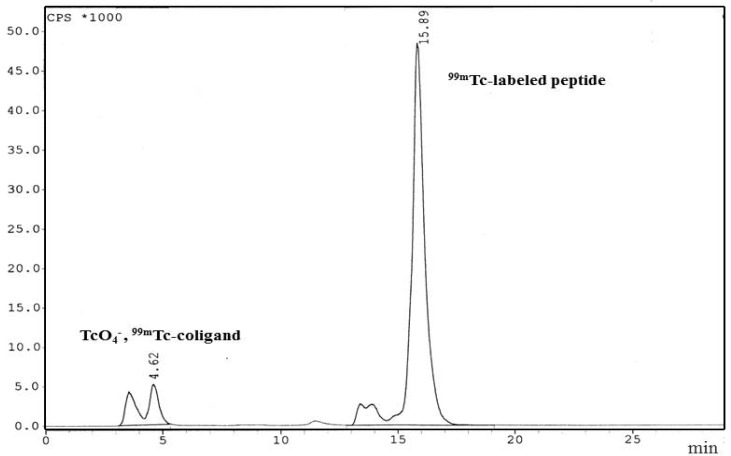
Reverse phase HPLC radiochromatogram of ^99^^m^Tc- labeled peptide

**Figure 3:**
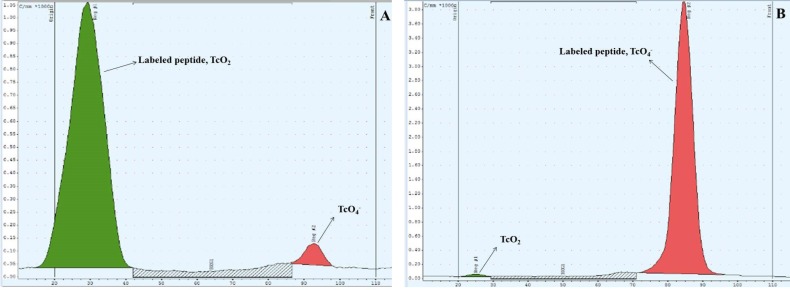
Radioactive-TLC chromatogram of labeled peptide using (A) ethyl methyl ketone and (B) acetonitrile 50% as solvent

**Figure 4:**
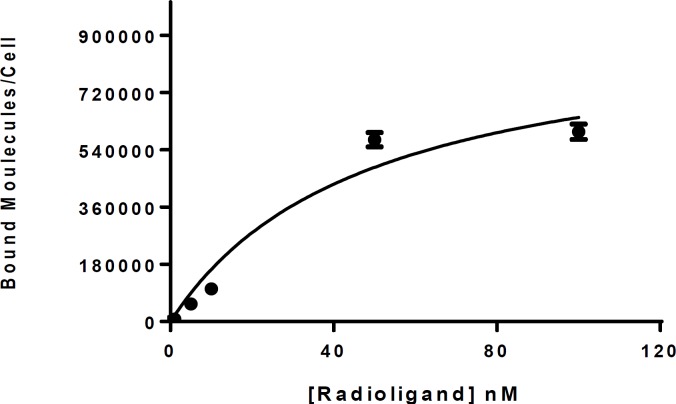
Saturation binding curve on B16-F10 cells which showed a K_d_ of 47.98±6.25 nM and a binding capacity of (B_max_= 950326±21531 bound molecules/cell)

**Figure 5:**
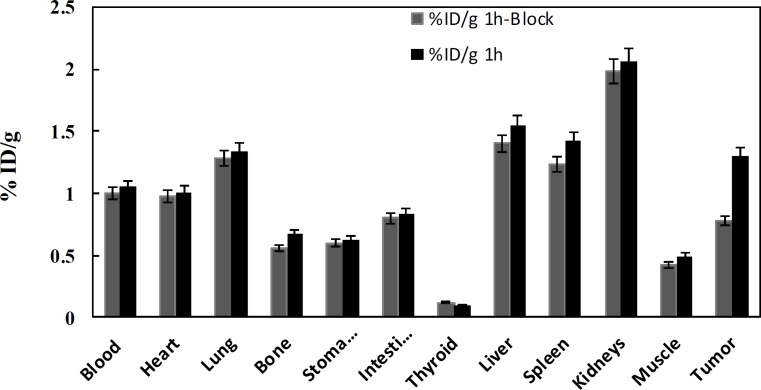
Biodistribution of labeled peptide in B16F10 tumor-bearing mice at 1 h post injection, unblocked (1 h) versus blocked (1 h) and *P<0.05

**Figure 6:**
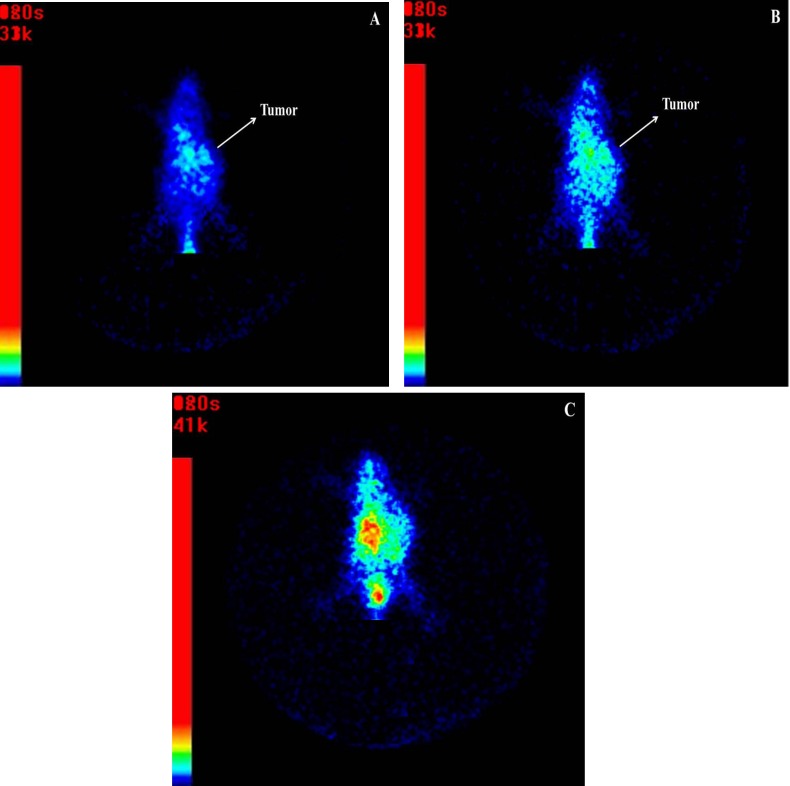
Anterior whole body images of mice bearing B16F10 tumor (A) 1 h, (B) 2 h and (C) 4 h after injection of ^99m^Tc-labeled peptide

***Radiolabeling with*** ^99m^ ***Tc and quality control***

Radiolabeling of HYNIC-peptide conjugate was performed by adding 20 µL of the stock peptide solution (5 µg of peptide) and 20 mg of tricine to water (0.5 mL) in a vial. 20 µL of stannous chloride dihydrate solution (2 mg/ml in 0.1 M HCl) was added along with 500 µL ^99m^Tc-pertechnetate (370-1480 MBq) in saline. The pH was adjusted to 7 by adding 1 M NaOH. The solution was incubated at room temperature for 10 min. Reaction mixtures were analyzed by RTLC and HPLC. RTLC was performed by use of silica gel plates which were developed in ethyl methyl ketone (^99m^Tc-pertechnetate R_f_=1.0), water: acetonitrile (1:1) (^99m^Tc-colloid R_f_=0.0) and sodium citrate 0.1 N with pH 5 to determine non-peptide-bound ^99m^Tc co-ligand and ^99m^Tc-pertechnetate with R_f_=1.0. The radioactivity was quantified by cutting the strip (1.5×10 cm^2^) into 1 cm pieces and counting in a well type gamma counter.

***Cellular and saturation binding***

B16F10 tumor cell line was used in this study purchased from Pasture Institute of Iran. The cells were cultured into a flask containing RPMI-1640 medium with 10% fetal bovine serum (FBS), 2 mM L-glutamine, penicillin G (100 U/ml) and streptomycin (100 µg/ml). The cells were seeded up to 2.5×10^6^ cells allowed to attach for 24 h at 37 °C in a humidified 5% CO_2_ atmosphere. Then the cells were washed with phosphate buffer saline (PBS 1x, pH=7.2) and incubated with a 100 µl (2.5 pmol, 140 KBq) of freshly radiolabeled peptide for 1 h. Also blocking test was carried out by using of excess cold peptide in concentration of 100 μmol/l. After incubation, the cells were washed three times with PBS to remove non bound radiotracer and then were removed. The cells harvested and washed for 10 minutes by addition of NaOH solution. The cellular bound and free radioactivity was measured in a well type Nal (Tl) scitinallation counter. The uptake (counts per minutes) was expressed as the percentage of total radioactivity. 

For saturation binding study, radio-complex with increasing concentrations (0.1, 0.5, 1, 5, 10, 50, 100 nM) was added to cell wells and incubated for 2 h at 37 °C. Blocking test was carried out by using of labeled peptide and cold peptide together, in concentration of 100 μmol L^-1^. After incubation, the cells were washed three times with phosphate buffer saline (PBS 1x, pH=7.2) and then were removed by addition of NaOH solution. The binding data were analyzed with non-linear regression with using Prism 5 software (Version 5.04, 2010).

***Biodistribution study in tumor bearing mice***

All animal experiments were carried out in compliance with the regulations of our institution that related to the performance of animal experiments. C57BL/6 mice were purchased from Pasture Institute of Iran. Groups of five mice (25-30 g, 8-10 weeks) were inoculated with 10 × 10^6^ B16F10 cells subcutaneously in 100 μL of medium into the right shoulder of each mouse. After 10-14 days, the tumors developed, and then labeled-peptide (20 MBq, 0.5 µg in 100 μL) was injected via a tail vein. As a blocking experiment, co-injection of radio-complex with cold peptide (100 μg) was performed for a group of five mice. After 30 min, 1, 2, 4 and 24 h post- injection, the mice were sacrificed and the organs of interest were excised, weighed, washed with normal saline and counted in the gamma counter. The results are presented as percent injected dose per gram tissue (%ID/g).

***Scintigraphy studies***

Imaging was separately achieved at times 30 min, 1, 2, 4 h post-injection of labeled peptide for tumor bearing mice. Before the imaging, mice were anesthetized with 0.05 mL ketamine 10% (3.3 mg) and 0.05 mL xylazine 2% (1.33 mg) intra-peritoneal. After about 5 min the animals were stabilized on a board in the posterior position and imaging was performed using a single head gamma camera (small area mobile, 140 keV, Siemens, Germany) equipped with high sensitivity parallel whole collimator and 10% window around 140 keV. Whole body image has been taken using a 256×256 matrix size with 5000 k counts at various time post injection. For getting an image, a 10% acceptance window around the 140 keV photo peak was used. The activity of regions of interest was specified from a serial gamma camera images recorded in animals with melanoma tumor.

***Statistical analysis***

Statistical evaluation was carried out by using the SPSS 11.5 version for windows. All results were expressed as mean values±SD (n=5) and statistical significance was determined as p<0.05.

## Results

***Radiolabeling and quality control***

A HYNIC-coupled cyclic structure of MccJ25 analog [HYNIC-*cyclo *(Cys^1^-Gly^2^-Ala^3^-Gly^4^-His^5^-Val^6^-Pro^7^-Cys^8^)-Tyr^9^-D-Tyr^10^-GABA^11^-D-Phe^12^-yr^13^-Gly^14^] was developed with the maximum yield of 40% and purity 98.8%. The chemical structure of conjugated peptide schematically was illustrated in Figure 1. The HYNIC-peptide conjugate was characterized by analytical reversed phase HPLC and ESI-MS (Table 1). Calculated mass for this new analog is 1653.65 g/mol and LC-MS analysis confirmed a [M+H]+ molecular ion of 1654.5, [M+Na]+ molecular ion of 1676.2 and [M+CH3CN+H]+ molecular ion of 1696.4. The specific radioactivity of the ^99m^Tc-HYNIC-peptide was 125±62 MBq/μmol while radiolabeling yield was >95% (n=3) as determined by HPLC and also RTLC. HPLC radiochromatogram was presented in Figure 2. A major peak at retention time of 15.89 min was related to ^99m^Tc-labeled peptide and free Tc-99m pertechnetate (^99m^TcO4^-^) and other impurities showed a minor peak at retention time of 4.62 min. The radiochemical impurities ^99m^Tc-pertechnetate (<1.0%), ^99m^Tc-radiocolloid (<2.0%), and ^99m^Tc-coligands (<1.0%) were negligible as determined by RTLC. As shown in Figure 3A, ethyl methyl ketone was used to check the percent of ^99m^TcO_4_^-^, which moved with the solvent front (R_f_=1.0), while other species remained at the point of spotting (R_f_=0.20). Acetonitrile/ water (1:1) was used as a developing solvent for another paper strip to determine the percent of reduced ^99m^Tc colloid species, which stayed at the origin (R_f_=0.16), while ^99m^Tc-labeled peptide and ^99m^TcO_4_^-^ moved at the top of the paper (R_f _=0.83) (Figure 3B).

***Cellular binding***

Affinity of labeled peptide to cellular membrane of B16F10 cells was assessed with saturation method. The obtained results of in vitro cell binding in presence of excess amount of unlabeled peptide as a competitor showed that radio-comoplex was taken up 30.74±0.5% to 2.5×10^6^ of B16F10 cells after 1 h contact and incubation at 37 °C. Saturation binding experiments showed affinity of radiotracer for B16-F10 cells (K_d_=47.98±6.25 nM) and binding capacity (B_max_=950326 ± 21531 bound molecules/cell) (Figure 4).

***Biodistribution***

The biological distribution of labeled peptide in B16F10 tumor-bearing nude mice at different time intervals post injection was illustrated in Table 2. The labeled peptide was rapidly removed from blood circulation with mean value of 1.64±0.03 %ID/g at 30 min to 0.48±0.09 %ID/g at 4 h post injection. The kidneys uptake value was 1.91±0.65 %ID/g at 30 min and this amount reached to 2.03±0.08 %ID/g at 2 h while decreased to 1.55±0.20 %ID/g at 4 h. The value of liver uptake was 1.67±0.05 %ID/g at early time which was lower than that of kidneys at this time. However, the high activity in kidneys and then liver suggests that the urinary system and hepatobiliary are as major route of excretion of radioactivity. The highest uptake of labeled peptide in melanoma tumor was observed at 1 h post injection (1.30±0.12 %ID/g) whereas, its uptake gradually decreased to mean value 0.80±0.30 %ID/g up 4 h after injection. By blocking the receptor through prior injection of cold peptide at 1 h, the uptake in tumor decreased to 40% of total tumor uptake (0.78±0.01 %ID/g versus 1.30±0.12 %ID/g), and this decline was not significant in the other organs (Figure 5).

***Tumor imaging***

Typical scintigrams at 1, 2 and 4 h after the injection of ^99m^Tc-labeled peptide in tumorized mice were shown in Figure 6 (A), 6 (B) and 6 (C) respectively. Hot spots in all images showed that labeled peptide was significantly taken in tumor and further whole body clearance of labeled peptide was occurred through renal and hepatobiliary systems.

## Discussion

Over the years, peptides hold great promise for clinical applications such as cancer diagnosis and therapy. Combination of cancer treatments to tumor specific peptides improves their therapeutic efficiency. Among those, antimicrobial peptides offer a great alternative for the conventional antibiotics against bacterial resistance and also display selective anticancer activities with low toxicity (2, 8 and 9).

In scintigraphy imaging, ^99m^Tc remains the radionuclide of choice because of its optimal nuclear properties (6.02 h half-life with 140 keV gamma photons) and availability via generators (17). High specific activity of ^99m^Tc labeling can be obtained by using bifunctional chelating agents. The most widely used bifunctional chelating agent is HYNIC (18). HYNIC-peptide labeling was performed employing tricine with high radiochemical purity (>95%, n=3).

The labeled peptide showed rapid and favorable binding to B16F10 melanoma cells at 37°C. The melanocortin 1 receptor (MC1R) that is overexpressed in most murine and human melanoma metastases is an attractive molecular target for imaging and radiotherapy of melanomas. In recent years, alpha-melanocyte-stimulating hormone (α-MSH) peptide analogs were prepared and evaluated for both melanoma imaging and radiotherapy (19, 20). While a variety of tumor imaging probes have been developed based on peptide receptor targeting, tumor cells binding of antimicrobial peptides could be related to specifically targeting cancer cells through the electrostatic attraction between the negatively charged components of the surface of the cells and the positively charged labeled antimicrobial peptide (21).

Biodistribution analysis was performed by injection of ^99m^Tc- labeled peptide (0.5 µg in 100 μL) to mice which were inoculated subcutaneously B16F10 cells. The biodistribution studies showed a low uptake of labeled peptide in normal muscle and the uptake levels continued to decrease further with time, leading to significantly reduced radioactivity levels in normal muscle. However a maximum of tumor/muscle uptake ratio (3.57±0.01) (Table 2) was obtained up to 2 h post injection. In an image study of melanoma bearing mice at 1 h post injection revealed that tumor uptake at early time after injection is high and decreases in a time dependent manner.

## Conclusion

In the present study, the conjugate of HYNIC-peptide was indirectly labeled with ^99m^Tc in presence of tricine as coligand with high radiochemical purity. The radio-complex was evaluated for melanoma tumor targeting and imaging; while the labeled peptide was excreted by renal and hepatobiliary routs. The tumor location could be visualized through scintigraphy imaging within 1 h post injection. Our results suggest that labeled antimicrobial peptides could be considered as a new category of radiotracer for diagnostic of tumor especially melanoma tumor.
